# Stress, Illness Perceptions, Behaviors, and Healing in Venous Leg Ulcers: Findings From a Prospective Observational Study

**DOI:** 10.1097/PSY.0000000000000436

**Published:** 2017-06-05

**Authors:** Jessica Walburn, John Weinman, Sam Norton, Matthew Hankins, Karen Dawe, Bolatito Banjoko, Kavita Vedhara

**Affiliations:** From the Institute of Pharmaceutical Science (Walburn, Weinman), Institute of Psychiatry, Psychology and Neuroscience, (Norton) King's College London; IMS Health (Hankins); School of Social and Community Medicine (Dawe), Faculty of Medicine and Dentistry (Banjoko), University of Bristol; and Division of Primary Care, School of Medicine (Vedhara), University of Nottingham, United Kingdom.

**Keywords:** adherence, depression, educational attainment, illness perceptions, stress, wound healing, **AQ** = Adherence Questionnaire, **HADS** = The Hospital Anxiety and Depression Scale, **HBQ** = Health Behaviour Questionnaire, **HR** = hazard ratio, **HRQoL** = health-related quality of life, **IPQ-R** = Illness Perception Questionnaire-Revised, **IPs** = illness perceptions, **IQR** = interquartile range, **M** = mean, **MCS** = Mental Component Summary, **PCS** = Physical Component Summary, **PSS** = The Perceived Stress Scale, **SD** = standard deviation, **SRM** = Self-Regulatory Model, **VLU** = venous leg ulcer

## Abstract

**Objective:**

The aim of the study was to investigate the impact of stress, illness perceptions, and behaviors on healing of venous leg ulcers.

**Methods:**

A prospective observational study of 63 individuals for 24 weeks investigated possible psychosocial predictors of healing. There were two indices of healing: rate of change in ulcer area and number of weeks to heal. Psychological variables were assessed at baseline using self-report measures (Perceived Stress Scale, Hospital Anxiety and Depression Scale, Revised Illness Perception Questionnaire, adapted Summary of Diabetes Self-Care Activities, Adherence Questionnaire, and Short-Form Health Survey).

**Results:**

Controlling for sociodemographic and clinical variables, for the 24 weeks, a slower rate of change in ulcer area was predicted by greater stress (standardized β = −0.61, *p* = .008), depression (standardized β = −0.51, *p* = .039), and holding negative perceptions or beliefs about the ulcer (standardized β = −1.4, *p* = .045). By 24 weeks, 69% of ulcers had closed. A more negative emotional response to the ulcer at baseline (i.e., emotional representation of the ulcer) was associated with a greater number of weeks to heal (hazard ratio [HR] = 0.63, 95% confidence interval [CI] = 0.41-0.95, *p* = .028). Higher educational attainment (HR = 3.22, 95% CI = 1.37–7.55, *p* = .007) and better adherence to compression bandaging (HR = 1.41, 95% CI = 1.06–1.88, *p* = .019) were associated with fewer weeks to heal. No other psychosocial variable (stress, perceptions about the ulcer, health behaviors) predicted weeks to heal.

**Conclusions:**

Alongside ulcer-related predictors, psychological and sociodemographic factors were associated with healing. Future research should explore mediating mechanisms underlying these associations and develop interventions to target these variables.

## INTRODUCTION

Venous leg ulcers (VLUs) are a common, recurrent chronic wound, which increase in prevalence with age (1). They have a significant impact on quality of life because they can be painful, malodorous, and exuding (2). The primary treatment is compression bandaging (3), and although this is effective, there is considerable variance in healing times (4). The search for predictors of healing speed has focused on clinical factors such as size and duration of the ulcer (5). However, a significant amount of variance remains unexplained, suggesting that other factors may be involved.

The association between stress and impaired healing has consistent empirical support in acute experimental wounds (6). Stress may directly affect healing via physiological changes associated with the stress response dysregulating immune function or indirectly as a result of unhealthy behaviors (7). A growing number of studies have shown that this relationship has validity outside the laboratory, reporting that stress impairs healing in postsurgical wounds (8). There is a lack of research in populations with chronic clinical wounds, which have an underlying pathology and where, by definition, healing is delayed. Previous studies on VLUs have reported a negative relationship between ulcer healing and negative emotions (9,10). However, these studies were limited by the use of cross-sectional designs and ratings of healing based on clinical judgement rather than objective measures. Nevertheless, the findings are consistent with recent prospective studies indicating depression to be associated with impaired foot ulcer healing (11,12).

A substantial literature exists demonstrating the importance of illness perceptions (IPs) in adjustment to chronic conditions (13) and clinical outcomes, including mortality (14). The common-sense Self-Regulatory Model (SRM) of Leventhal encompasses both cognitive and emotional representations of illness, which can influence illness outcomes via a range of behavioral and emotional coping responses. Although no study has applied the SRM framework to investigate wound healing in VLUs, there is evidence showing that IPs are associated with speed of healing of postoperative oral wounds (15). Furthermore, beliefs about symptoms, understanding of ulceration and personal control beliefs, have been found to be predictive of self-care behaviors in chronic foot ulcers (16). Interventions in other chronic conditions show that these perceptions are modifiable predictors of health outcome (17).

Despite the fact that patients with VLUs are encouraged to maintain a healthy life-style, there is little evidence demonstrating an association between life-style factors and healing rates in this population (3). A recent trial of VLU patients found that a life-style counselling intervention increased physical activity and reduced number of “wound days” for 18 months (18). Evidence indicates that adherence to treatment is often suboptimal (19), but relatively little is known about the effects of this on healing.

Research on the role of psychosocial factors in VLU healing is scarce and limited by lack of prospective designs, poor control of potentially confounding clinical characteristics, and failure to use quantitative measures of healing. The following study addressed these limitations by using a prospective design to explore the impact of stress, IPs, and a range of behavioral factors on healing, while controlling for ulcer characteristics, comorbidity, and sociodemographic variables. It was hypothesized that psychological stress, IPs, and health behaviors would be associated with impaired healing defined as a slower rate of change in ulcer area and a greater number of weeks to heal.

## METHOD

### Design

A prospective observational study assessed the relationship between potential healing predictors measured at baseline (study entry) and ulcer status at 6-, 12-, and 24-week follow-up.

### Participants

Patients were recruited from primary care clinics (see hereafter) and eligible if they were diagnosed with a VLU, ankle/brachial pressure index of 8.0 to 1.3 inclusive, able to walk, and tolerate compression bandaging. Patients were excluded if they had type 1/2 diabetes, a current major comorbidity (e.g., cancer, myocardial infarction) or one that had occurred in the previous 12 months, were undergoing treatment with platelet-derived growth factor or tissue-engineered skin, unable to speak, and read or write in English.

Of the 88 eligible, 22 patients chose not to participate and three patients were excluded because of misdiagnosis. Sixty-three patients completed baseline ulcer assessment and 60 returned self- report questionnaires. Attrition was minimal; two participants (3%) left the study by 6 weeks, and a further three participants (5%) by 24 weeks. Participants who left the study and whose ulcer status was not known differed in terms of having ulcers of longer duration at study entry (median = 68 weeks, median = 16 weeks, *U* = 98.00, *p* = .013). There were no clinic-specific baseline differences.

A priori power calculation was based on multiple linear regression analysis to detect a significant effect of stress on rate of change in ulcer area. The effect size was informed by a meta-analysis of 11 studies investigating the relationship between stress and wound healing across clinical and experimental wound types (6), which estimated that stress would be responsible for 16% of the variance in the rate of healing. Using this estimate (assuming α = 0.05, power at 80%, six control variables), a sample size of at least 44 participants was required to detect an effect size of *f*^2^ value of 0.19 (equivalent to *R*^2^ = 0.16). A target sample size of 100 within a recruitment window of 45 months (March 2006-December 2009) was set. This was based on the power calculation and intention of conducting exploratory survival analysis (weeks to healing).

### Procedure

After ethical approval by East Kent Local Research Ethics Committee, a consecutive sample of patients attending one of five primary care leg ulcer outpatients clinics in the London area were invited to participate. Eligible patients were identified from medical records and approached while waiting for routine appointments. After giving informed consent, patients entered the study at the next routine appointment (1–2 weeks after provision of study information) when ulcer baseline measurement (week 0) was completed by the nurse. Ulcer history was recorded from medical notes. Participants were given the questionnaire to complete at home and returned it by post or at next routine weekly/biweekly appointment. At each study follow-up (6, 12, 24 weeks), the ulcer was measured by the nurse providing care on that day, if the ulcer had healed the healing date was recorded.

### Measures

#### Primary Outcome

This study assessed change in the VISITRAK (Smith + Nephew, London, United Kingdom) measurement of surface ulcer area (square centimeter), as a quantitative, standardized, and robust index of healing. The nurse providing routine care traced the ulcer that was transferred to VISITRAK by the researcher to calculate the area, by retracing the outline with an electronic stylus. A convenience sample of 26 tracings at week 6 were assessed by a second researcher. Interrater reliability was high (intraclass correlation = .99, 95% confidence interval [CI] = 0.99–1.00). Both nurses and researchers were blind to the predictor scores. For analysis, healing was expressed in terms of the rate of change in ulcer area observed across the four assessments (i.e., square centimeter per week).

#### Secondary Outcome

Ulcer area alone would not capture a clinically relevant outcome from the patient's perspective. Therefore, the number of weeks for the ulcer to heal from study entry up to 24 weeks was recorded. The nurse decided if the ulcer had healed on the basis of clinical judgement.

#### Baseline Predictor Variables (Self-report)

##### Sociodemographic Factors

Sex, age, and educational attainment (passing the first level of school public examination, e.g., General Certificate of Secondary Education, United Kingdom, currently taken at 16 years) were recorded.

##### Clinical and Ulcer Characteristics

Ulcer area (square centimeter) at baseline, ulcer duration (weeks), previous episodes of ulceration, and number of comorbid conditions (self-report) were recorded.

##### Psychosocial and Behavioral Factors

###### Stress

1. The Perceived Stress Scale (20) contains 14 statements asking participants how frequently they felt a certain way in the previous 4 weeks, by selecting from a five-item Likert scale (0–4) ranging from “never” to “very often.” Scores range from 0 to 56, where a higher score indicates greater perceived stress (Cronbach α = 0.87, current study).
2. The Hospital Anxiety and Depression Scale (21) was used to assess levels of anxiety and depression. The HADS is a brief, self-report questionnaire containing seven statements about anxiety and depression. Scores for subscale totals range from 0 to 21. It is a widely used scale, and internal reliability for both subscales was high (depression α = 0.80, anxiety α = 0.84).

###### Illness Perceptions

An adapted version of the Illness Perception Questionnaire-Revised (IPQ-R, 22) was used to assess participants' perceptions of their VLU. It includes nine subscales assessing perceptions about illness identity (number of symptoms attributed to the illness), cause of illness (not reported here), timeline (duration, cyclical), consequences, control/cure (by own actions and medical treatment), negative emotional representation of ulcer, and illness coherence. All subscales, apart from symptom identity, required responses to statements on a five-point Likert scale. Mean score was calculated to enable comparison across subscales (1–5), a higher score indicates stronger belief. Internal reliability for subscales was acceptable (Cronbach α: timeline - duration, 0.86; cyclical, 0.83; personal control, 0.71; treatment control, 0.68; consequences, 0.86; coherence, 0.85; emotional representation, 0.86. Symptom identity was assessed by summing symptoms identified by patients as VLU related from a list of 14 general nonspecific symptoms.

Cognitive IPs, excluding negative emotional representation, were grouped using cluster analysis to reflect that SRM proposes that people hold coherent schema of interacting perceptions (23). K-means clustering (24) resulted in two groups: a *negative* illness schema dominated by negative beliefs about the ulcer and a *sanguine* illness schema typified by more optimistic beliefs. Further details are provided in the lead author's doctoral thesis (25) with the groupings consistent with previous research in other conditions (26).

###### Health Behaviors

An adapted version of the Summary of Diabetes Self-Care Activities Measure (27) and a Health Behavior Scale (28) was used to measure diet (fruit and vegetable, fat consumption), physical activity level (general activity or organized exercise), sleep (average hours per night), as well as smoking and alcohol consumption (number of cigarettes smoked/drinks consumed). Unless otherwise stated, the number of days (1–7) during the previous week when they participated in each behavior was recorded. Alcohol consumption was converted into units to identify intake greater than recommended daily levels (29) translated into weekly intake (>21 U for men, >14 U for women).

###### Adherence to Treatment

A questionnaire was designed to assess adherence to compression bandaging and leg elevation. Participants recorded the number of days when they had not worn bandages and whether they adjusted their bandages (i.e., cutting or loosening; 1 = yes/0 = no) during the previous week. Frequency of leg elevation was measured on a five-point Likert scale (*1 = legs were elevated all the time when seated, 5 = never elevated when seated*) during the previous week. The compression bandaging items were summed, producing a score of 0 to 8. Scores were reversed, so a higher score indicated higher adherence.

###### Health-Related Quality of Life

As an indication of overall physical and emotional well-being, the Short Form Health Survey-12 (30) was completed. It has 12 items that assess eight health-related quality of life (HRQoL) dimensions, and responses are recorded on Likert scales. One dimension measures bodily pain, an important component of HRQoL for those with a painful ulcer. The eight domains are reduced to two summary domains “Physical Component Summary” (PCS) and “Mental Component Summary” (MCS), by summing weighted standardized scores from four domains, respectively. Higher scores indicate higher HRQoL. Both dimensions had acceptable internal reliability (PCS α = 0.79, MCS α = 0.74).

### Statistical Analyses

#### Rate of Change in Ulcer Area

Linear mixed-effects models were estimated to determine predictors of the rate of change in ulcer area from baseline to 24 weeks. This approach allowed for simultaneous examination of the cross-sectional and longitudinal associations between the psychological and behavioral factors with ulcer area. Ulcer area at baseline, 6, 12, and 24 weeks for each individual was entered as the outcome variable. A random intercept accounted for the nonindependence of the outcome variable within individuals and allowed ulcer area at baseline to vary across individuals. A random linear slope for time allowed the rate of change to vary across individuals. Because time in weeks from baseline for each assessment was entered as a continuous variable, its coefficient is interpreted as the expected difference in ulcer area for each additional week following the baseline assessment (i.e., the average weekly change in ulcer area between baseline and 24 weeks). A quadratic time slope allowed for a nonlinear rate of change in area. A random effect for the quadratic time slope was not included because preliminary analysis indicated that the estimated variance was close to zero. Predictor variables were included in the model allowing for determination of the cross-sectional association between the predictors and ulcer area at baseline. Interaction terms for the predictor variables with the linear time slope allowed for the determination of the association of the predictor and the rate of change in ulcer area (i.e., predictors of change in ulcer area were identified while controlling for baseline associations with ulcer area). Separate models were estimated for each psychological and behavioral variable. All models controlled for sociodemographic factors (age, sex, educational attainment), comorbidity, ulcer characteristics (ulcer duration, first ulcer, plus area at baseline for the rate of change in ulcer area), and PCS HRQoL at baseline.

#### Time Taken for Ulcer to Heal From Baseline to 24 Weeks

Time to ulcer healing was examined using Cox proportional hazards regression survival models. The outcome variable was the time in days from the baseline until the ulcer healed, which was censored at 24 weeks where the ulcer had not healed by that time. Separate models assessed the hazard ratio (HR) for each psychological and behavioral variable controlling for sociodemographic factors, comorbidity, ulcer characteristics, and PCS HRQoL.

## RESULTS

Baseline sample characteristics are displayed in Table 1. A total of 60.3% were female, mean (M) age was 68.1 years, and 44.4% achieved first level of school examinations. Mean ulcer area was 11.5 cm^2^, and 61.9% had a previous episode of ulceration. Most (71.0%) reported having at least one comorbid condition, with osteoarthritis (41.3%) and high blood pressure (33.3%) being the most common. Based on the survival models accounting for censoring, the median time to heal was 12 weeks. At 24 weeks, 30.4% of ulcers remained open. Participants had lower physical and mental health-related quality of life summary scores compared with the general population (United States, M = 50; 30). Few participants scored above the threshold (>7) suggestive of emotional disturbance for anxiety (30.2%) and depression (22.2%). More than a third had a negative emotional representation of ulcer (39.0%), scoring above the midpoint. Baseline behavioral variables show that adherence to compression bandaging was high with more than 80% not removing the bandage and more than 70% not adjusting it. Leg elevation was less well adhered (60.3% elevating leg “*sometimes*,” “*occasionally*,” or “*never*”*).* Few smoked in the previous week (20.6%) and consumed alcohol above the recommended levels (23.8%).

**TABLE 1:**
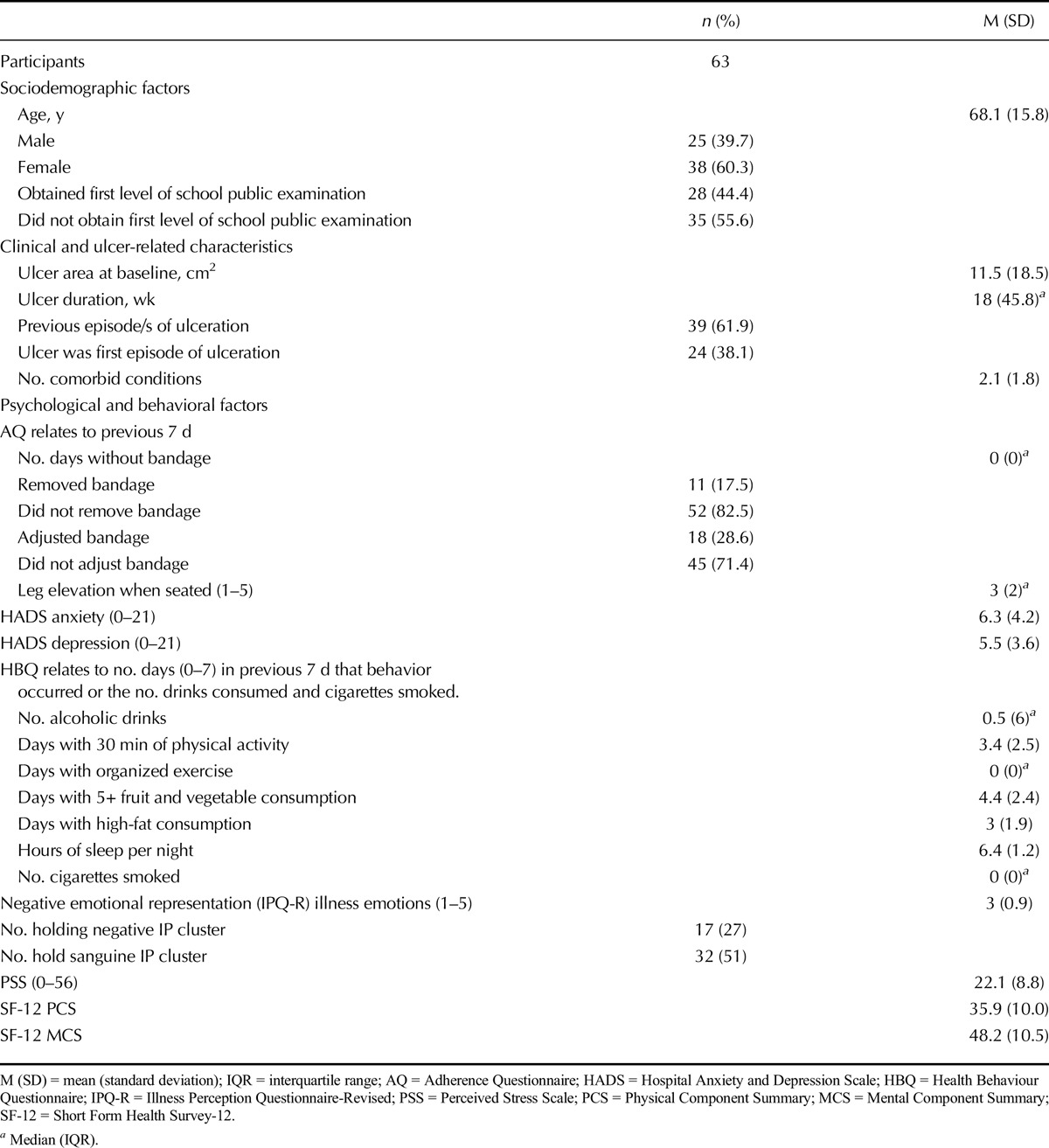
Baseline Characteristics of the Sample

In comparison with those with sanguine perceptions, participants with a negative cluster reported a greater number of ulcer symptoms (M [standard deviation {SD}] = 4.1 [2.0] *negative*, 1.5 [1.1] *sanguine*, *t*(47) = 5.1, *p* < .001) and believed the ulcer to be a chronic condition (3.1 [0.8], 2.6 [0.7], *t*(47) = 2.3, *p* = .024). They thought that it was a cyclical condition (3.3 [0.8], 2.7 [1.0], *t*(47) = 2.2, *p* = .035) with serious consequences (3.7 [0.7], 2.6 [0.7], *t*(47) = 5.1, *p* < .001). There was little difference between the clusters regarding personal control over the ulcer (3.3 [0.6], 3.5 [0.7], *t*(47) = −0.9, *p* = .40), confidence in the compression bandaging to control their ulcer (4.2 [0.7], 4.0 [0.5], *t*(47) = 0.7, *p* = .50), and both thought that they had an adequate grasp of the condition (3.6 [0.8], 3.3 [0.1], *t*(47) = 1.5, *p* = .14). In summary, those with the sanguine schema perceived the ulcer to be acute, with fewer symptoms and less serious consequences. Participants holding the negative cluster of cognitive IPs exhibited a more negative emotional representation of the ulcer (3.7 [0.9], 2.7 [0.8], *t*(47) = 4.1, *p* < .001).

### Preliminary Analyses

#### Rate of Change in Ulcer Area From Baseline to 24 Weeks

**FIGURE 1:**
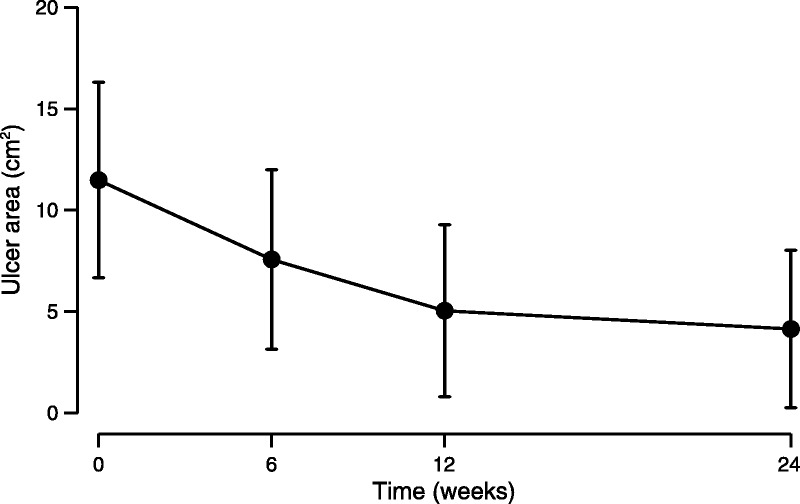
Estimated mean ulcer area (square centimeter) between 0 and 24 weeks with 95% confidence interval limits.

Initial analysis indicated the best-fitting mixed-effects model, describing the shape of the ulcer area healing trajectory to include linear and quadratic time slopes. This allowed for nonlinear (decelerating) change in ulcer area over time. The model indicated that ulcer area decreased at a rate of .8 cm^2^ per week (SD = .2), which decelerated by .02 cm^2^ per month. No ulcer increased in size (Fig. 1). A model (model 1) was estimated, including sociodemographic, comorbidity, ulcer-related factors, HRQoL PCS, and the interaction with the random time slope (Table 2). This allowed for the simultaneous estimation of the association between the predictors with ulcer area at baseline and the weekly rate of change in ulcer area. Negative unstandardized regression coefficients *b* for the change ulcer area indicate slower healing, where the rate is square centimeter per week. Younger age, being female, longer ulcer duration, and higher comorbidity were related to a larger ulcer at baseline. Younger age, longer ulcer duration, having had previous ulcers, and worse physical quality of life were related to slower rates of healing.

**TABLE 2:**
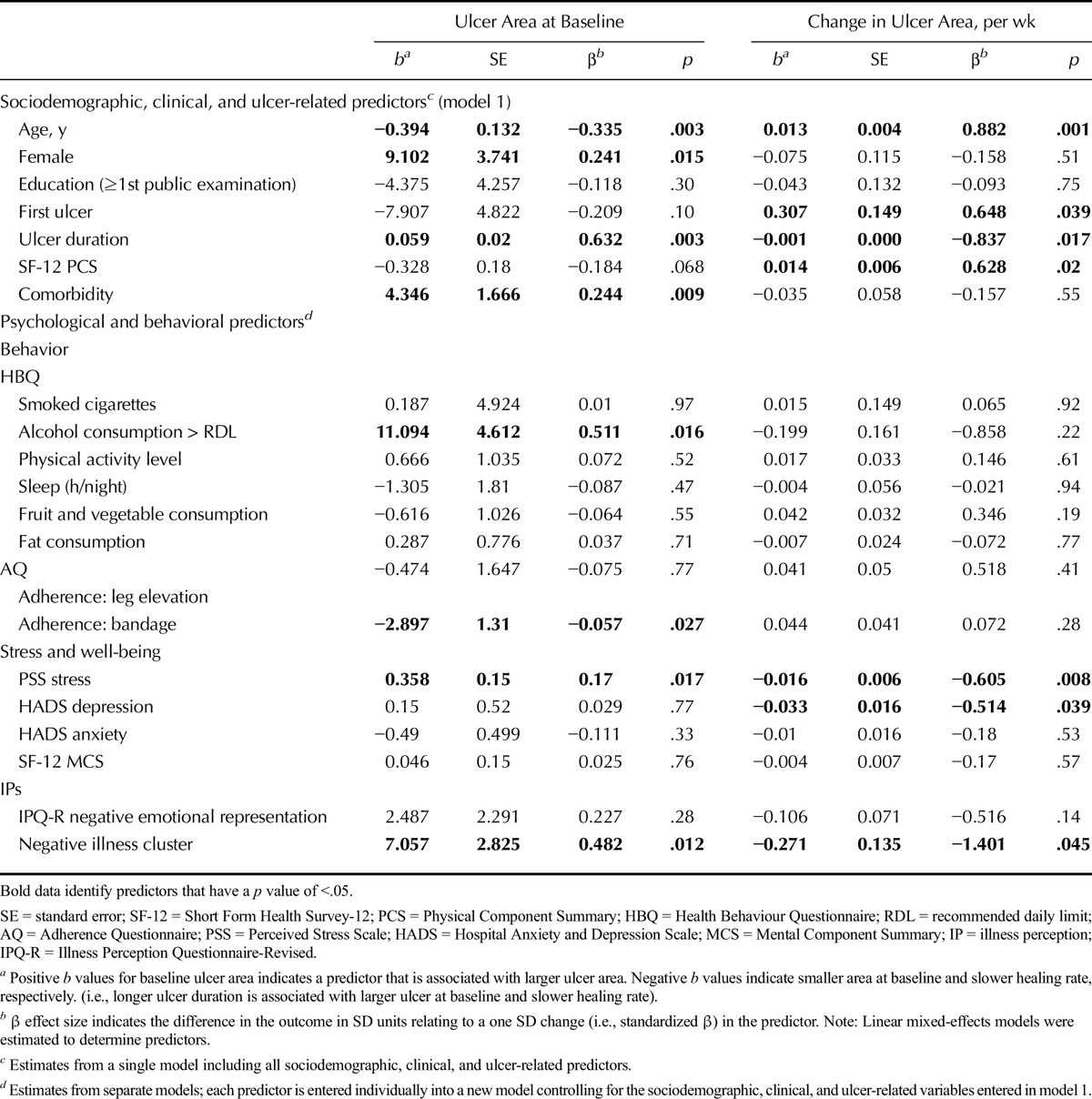
Psychological and Behavioral Variables Associated With Ulcer Area at Baseline and Weekly Rate of Change in Ulcer Area, Controlling for Sociodemographic, Clinical, and Ulcer-Related Predictors

### Hypothesis Testing

#### Psychological Stress, IPs, and Health Behaviors Would be Associated With a Slower Rate of Change in Ulcer Area

To determine the association between the predictors with ulcer area at baseline and the weekly rate of change in ulcer area, separate mixed-effects models were estimated for each of the psychological and behavioral predictors, including the interaction of the predictor with the random time slope (Table 2). Controlling for the variables entered in model 1 (sociodemographic, comorbidity, ulcer-related factors, HRQoL PCS), higher levels of perceived stress, holding negative ulcer perceptions, poorer adherence to bandage, and higher alcohol consumption were related to larger ulcer area at baseline. Significantly slower rates of healing were observed for those with a negative cluster of IPs (β = −1.4, *p* = .045), higher perceived stress (*β* = −0.6, *p* = .008), and depression at baseline (*β* = −0.5, *p* = .039). No behavioral variables (adherence, alcohol consumption, diet, physical activity, smoking, sleep) were related to healing rate.

#### Psychological Stress, IPs, and Health Behaviors Would be Associated With a Greater Number of Weeks to Heal

A model including sociodemographic variables, comorbidity, ulcer variables, and HRQoL PCS (model 1) indicated that the probability of healing was greater for those with higher educational attainment (Table 3). Controlling for these variables, the probability of healing was lower for those with a strong negative emotional representation of the ulcer at baseline. The median healing time was 22 weeks compared with 11 weeks for those with a less negative representation. Better bandage adherence predicted fewer weeks to healing. The median healing time was 10 weeks increasing to 16 weeks for those with worse adherence.

**TABLE 3:**
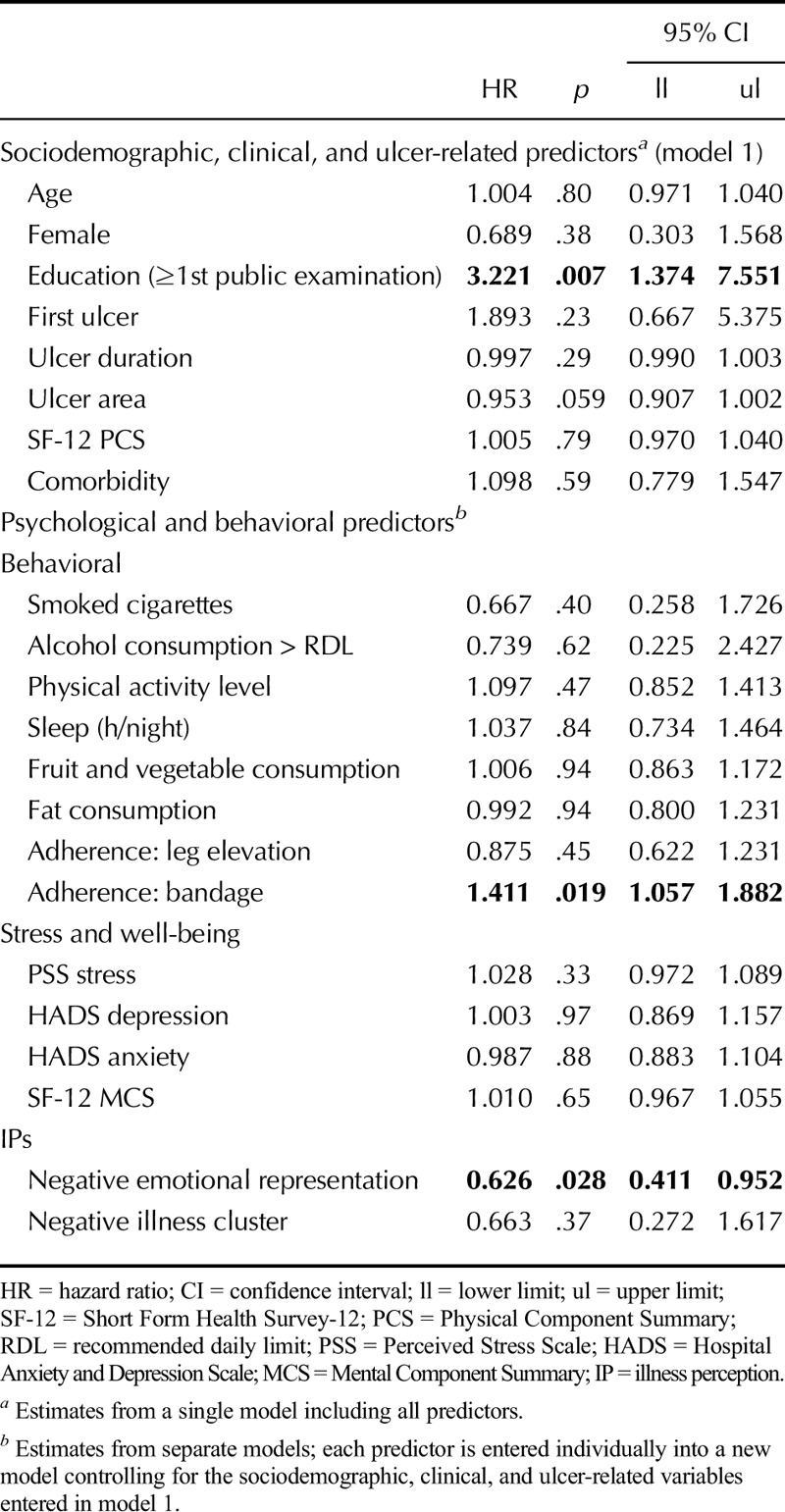
Cox Proportional Hazards Models of Predictors of Time Until Ulcer Healed (Weeks)

## DISCUSSION

Higher levels of stress, depression, and exhibiting a negative cluster of IPs were associated with a significantly slower rate of change in ulcer area, independent of sociodemographic, comorbidity, and ulcer characteristics. The size of the relationships indicated clinical relevance (i.e., meaningful to the patient or clinician) albeit that they were not significant when measuring the overall time taken for ulcers to heal, potentially because of lower power. Negative emotional representation of the ulcer predicted increased time to healing. Higher adherence to bandaging and higher educational attainment were associated with fewer weeks to heal. As anticipated, ulcer-related variables were associated with impaired healing.

### Rate of Change in Ulcer Area

That stress was associated with impaired healing is consistent with the findings of previous studies assessing chronic leg ulcers (9,10) and other chronic wounds (11). Because of the prospective design and control of baseline ulcer area, this study showed that stress was associated with having a worse wound *and* with impaired healing, a prerequisite of a causal connection. This replicates the findings of experimental wound studies with healthy populations (6) despite the inevitable “noise” of the multiple factors that could affect healing in a chronic clinical wound. Without the context of the existing stress and healing literature, there would be greater concern that potential confounding variables not measured in this study (e.g., extent of venous pathology, medications) could account for the stress healing relationship. Greater control of these variables would confirm the size of the association and measures of neuroendocrine, and immune markers would help identify mediating mechanisms.

This study adds to the growing body of evidence highlighting the role of IPs in influencing clinical outcomes, extending the finding of McCarthy et al. (15) to show that patient perceptions are associated with impaired healing in chronic wounds, as well as acute surgical wounds. Although having a larger ulcer was associated with negative IPs, this does not explain the relationship between IPs and healing because the analyses controlled for the relationship between IPs and ulcer area at baseline, as well as other ulcer-related predictors. This is consistent with the wider IP literature showing that IPs are not merely a function of clinical severity and are independent predictors of outcome across chronic conditions (13). Because it is likely that patients have a weekly update on the status of their wound at their clinic appointment, IPs will be influenced by this information indicating that the relationship between IPs and wound status is bidirectional. Future intervention studies could investigate this complex relationship by modifying IPs and measuring the impact on healing as well as exploring potential mediators (e.g., adherence, stress, health behaviors).

Although older age is a known predictor of slower wound healing (31), in our sample, being younger was associated with having a larger ulcer at baseline and a slower healing rate, suggesting that the relationship in VLUs is more complex. It is hypothesized that because VLUs are usually a disease of older age, if a younger person has an ulcer, this could be because they have a more serious venous pathology, which may then explain the relationships reported here.

### Time Taken for Ulcer to Heal

This is the first study, known to the authors, to report that low educational attainment is associated with impaired healing in VLUs. Low levels of educational attainment have been associated with a wide variety of poorer health outcomes, including life expectancy (32). There may be multiple factors responsible for the relationship, including low levels of health literacy (33). If attainment is a marker of lower socioeconomic status, these findings are consistent with previous research that reported a univariate association between delayed healing and lower socioeconomic status (34).

Interestingly, different measures of “stress” had varying relationships with healing. Negative emotional representation of the ulcer was a predictor of time taken to heal, whereas stress, depression, and anxiety were not. Moss-Morris and colleagues state that illness-specific emotional representation is not merely a reflection of general negative affect (22). It remains to be seen how distinct ulcer-specific emotional response is from general distress and whether it has a unique relationship with healing mediated by different mechanisms.

That higher adherence to compression bandaging was associated with fewer weeks to healing supports the view that good adherence is important for healing (35) and adds to the relatively sparse empirical data showing a relationship between adherence and healing (36). Although this does substantiate the view that a wound, which is not progressing, could indicate poor adherence, adherence to bandaging in the sample is high as has been reported elsewhere (37).

Variables that predicted healing for one outcome were not related to healing across both outcomes. It is logical that the statistically significant relationships identified for rate of change in ulcer area (e.g., stress variables) may not be strong enough to be statistically significant for a less powerful analysis based on weeks to heal. However, this would not explain why educational attainment was not related to the rate of healing, when it was a strong predictor of weeks to heal. This could be measurement error and an indication that this study failed to comprehensively capture the complex healing process, being limited to surface area only, and did not assess the balance of biomarkers, such as matrix metalloproteinase-9, as markers of healing (38) or ulcer depth. It is also acknowledged that despite being better controlled than earlier studies, the study did not control for potential confounding variables such as medication, weight, and venous pathology. Alternatively, different predictors may affect different aspects of VLU healing as has been shown in studies of healing in foot ulcers (11). Future studies should attempt to use more than surface measurement to gain a comprehensive picture of the healing process.

Stress accounted for approximately 8% of the variance of healing, after controlling for sociodemographic and ulcer characteristics. This equates to half the predicted effect size used to inform the sample size calculation, which was based on data from predominately acute experimental wounds (8 of 11 studies, 6) rather than comparable chronic wounds. The relationship between stress and healing in chronic wounds is likely to be more complex than in healthy participants with acute wounds, because they have an underlying pathology and a wound that has considerable impact on daily living. As a consequence, the study is potentially underpowered, which could explain the inconsistency across outcomes and meant unique variance explained by each psychosocial predictor, controlling for other predictors that could not be identified. The study was not powered for the secondary analysis, which will have contributed to the lack of statistically significant relationships between variables and time taken to heal, despite relatively large effect sizes.

Further research is needed to ascertain the relative importance of stress and IPs and to understand the interrelationships between them. To establish causal relationships, the next step is to design interventions targeting stress and negative IPs for patients with VLUs. Stress management interventions in acute wounds have been shown to improve healing in experimental (emotional disclosure, 39) and surgical wounds (brief relaxation, 40) compared with control. In light of the potential importance of educational achievement and of the modest educational attainment of the sample, clinicians should take steps to ensure that patients *fully* understand their condition and treatment recommendations.
